# Computational Challenges and Collaborative Projects in the NCI Quantitative Imaging Network

**DOI:** 10.18383/j.tom.2016.00265

**Published:** 2016-12

**Authors:** Keyvan Farahani, Jayashree Kalpathy-Cramer, Thomas L. Chenevert, Daniel L. Rubin, John J. Sunderland, Robert J. Nordstrom, John Buatti, Nola Hylton

**Affiliations:** 1 Cancer Imaging Program, National Cancer Institute, Bethesda, Maryland;; 2 Massachusetts General Hospital, Harvard Medical School, Boston, Massachusetts;; 3 Department of Radiology, University of Michigan, Ann Arbor, Michigan;; 4 Department of Radiology, Biomedical Data Science, and Medicine (Biomedical Informatics Research), Stanford University, Palo Alto, California;; 5 Department of Radiology, University of Iowa, Iowa City, Iowa;; 6 Department of Radiation Oncology, University of Iowa, Iowa City, Iowa; and; 7 Department of Radiology, University of California San Francisco, San Francisco, California

**Keywords:** quantitative imaging, cancer therapy, crowdsourcing, challenge, collaborative project

## Abstract

The Quantitative Imaging Network (QIN) of the National Cancer Institute (NCI) conducts research in development and validation of imaging tools and methods for predicting and evaluating clinical response to cancer therapy. Members of the network are involved in examining various imaging and image assessment parameters through network-wide cooperative projects. To more effectively use the cooperative power of the network in conducting computational challenges in benchmarking of tools and methods and collaborative projects in analytical assessment of imaging technologies, the QIN Challenge Task Force has developed policies and procedures to enhance the value of these activities by developing guidelines and leveraging NCI resources to help their administration and manage dissemination of results. Challenges and Collaborative Projects (CCPs) are further divided into technical and clinical CCPs. As the first NCI network to engage in CCPs, we anticipate a variety of CCPs to be conducted by QIN teams in the coming years. These will be aimed to benchmark advanced software tools for clinical decision support, explore new imaging biomarkers for therapeutic assessment, and establish consensus on a range of methods and protocols in support of the use of quantitative imaging to predict and assess response to cancer therapy.

## Introduction

The Quantitative Imaging Network (QIN), currently with 25 member institutions and supported by the National Cancer Institute (NCI), is engaged in research and development of quantitative imaging for predicting or evaluating response to cancer therapy. Projects in QIN address a range of cancers, including brain, head and neck, lung, breast, and prostate, among others, using advanced clinical imaging modalities, such as computed tomography (CT), positron emission tomography (PET/CT) and magnetic resonance imaging (MRI). A central aim is refinement and standardization of advanced techniques including dynamic contrast-enhanced MRI (DCE-MRI) and diffusion-weighted imaging for clinical use. QIN teams are multidisciplinary and include a wide range of expertise such as medical and radiation oncologists, radiologists, and imaging and data scientists. In addition to the research conducted at each member site, the network as a whole addresses a range of scientific and technical developments related to imaging acquisition, image annotation and markup, analysis, biomarker validation, and potential for deployment in prospective clinical trials. Currently, these are accomplished through several of the following QIN Work Groups:

(1) Data Acquisition (DA).
(2) Image Analysis and Performance Metrics (IAPM) and IAPM subgroups—MRI and PET/CT.
(3) Bioinformatics/IT and Data Sharing (BIDS).
(4) Clinical Trial Design and Development (CTDD).

The network environment provides an excellent opportunity for cooperative evaluation of imaging technologies, quantitative assessment of image analysis algorithms, and software (SW) tools developed by individual QIN teams (1). Moreover, since 2013, QIN teams have engaged in a variety of network-wide (multicenter) projects under the rubric of “challenges” (Table 1). These projects have involved the evaluation of SW tools, imaging biomarkers, and imaging evaluation of reference objects (phantoms) in support of broad network objectives. Several such completed projects have helped generate new knowledge and provide valuable insights into various approaches by QIN teams on specific scientific tasks. These efforts have also led to the publication of results and recommendations for best practices in quantitative imaging methods in oncology (2–5).

**Table 1.** QIN Performing Nation-wide Technical CCPs

Title	Description
Breast DCE-MRI	Evaluate variations in DCE-MRI assessment of breast cancer response to neoadjuvant chemotherapy caused by differences in software tools/algorithms used by different participating sites (2).
QIN ADC	Quantify differences in diffusion maps (4).
DCE-MRI Arterial Input Function	Assess stability of AIF across various informatics tools in patients with prostate sarcoma (3).
Lung CT Segmentation	Demonstrate stability of segmentations as functions of algorithms in patient studies and accuracy in a phantom (5).
FDG PET Segmentation	Quality and variability analysis of 3-dimensional FDG PET segmentations based on phantom and clinical data.
Breast MRI Metrics of Response (BMMR)	(a) Identify imaging metrics (predictors) deliverable from contrast-enhanced MRI acquired in ACRIN 6657 trial that show significant association with recurrence-free survival; and (b) demonstrate improvement in predictor performance over functional tumor volume.
Interval Change Using NLST Chest CT Scans	Remove algorithm bias as a confounder and instead compare algorithmic ability to detect segmentation change between 2 time points.
Dynamic PET-MISO	Assess accuracy/stability of tumor segmentation in Dynamic PET scans using FMISO.
CT Image Feature	Assess stability of features computed using different segmentation results.
DICOM Storage—Parameter Map Storage	Generate ADC maps in uniform DICOM format for diffusion phantom validation.
DSC MRI	Evaluate accuracy of single-echo DSC MRI algorithms to predict predetermined outcomes.
Validation of Gradient non-Linearity Bias Correction	Perform gradient non-linearity bias correction for independent DWI phantom measurements.

Abbreviations: QIN, Quantitative Imaging Network; CCPs, Challenges and Collaborative Projects; DCE-MRI, dynamic contrast-enhanced magnetic resonance imaging; ADC, apparent diffusion coefficient; AIF, arterial input function; CT, computed tomography; FDG PET, fluorodeoxygloucose positron emission tomography; MRI, magnetic resonance imaging; ACRIN, American College of Radiology Imaging Network; NLST, National Lung Screening Trial; PET-MISO, positron emission tomography-fluoromisonidazole; FMISO, fluoromisonidazole; DICOM, Digital Imaging and Communications in Medicine; DSC, dynamic contrast enhanced; DWI, diffusion-weighted imaging.

As an example, in a study of variations in DCE-MRI to evaluate breast cancer therapy response to neoadjuvant chemotherapy by Huang et al. (2), data acquired at one site from 10 patients were analyzed at 7 sites with 12 SW tools based on 3 different tracer kinetic models. They observed considerable variability between the various SW packages, estimated as the within-subject coefficient of variation values, for *K*^trans^, a rate constant for contrast agent plasma/interstitium transfer, and *v*_p_, the plasma volume fraction. This occurred despite providing a consistent region of interest and arterial input function (AIF). They found that parameter agreement improved when comparing algorithms based on the same kinetic model, and observed improved concordance in assessment of parameter percentage change compared with parameter absolute value. In another multicenter data analysis study, Huang et al. (3) assessed the impact of variations in AIF quantification on prostate DCE-MRI kinetic modeling and parameter estimation at 9 centers, using imaging data acquired from 11 patients at one center. They observed that assessment of AIF across sites improved when reference tissue adjustments were considered, causing a reduction in variations in *K*^trans^ and *v*_e_ (extravascular, extracellular volume fraction). They also found that the contrast agent intravasation rate constant, k_ep_ (= *K*^trans^/*v*_ep_), was less sensitive to AIF variations than *K*^trans^ alone, concluding that k_ep_ may be a more robust imaging biomarker for assessment of prostate microvasculature than *K*^trans^.

QIN centers have also performed collaborative analytical studies using data acquired from phantoms to characterize platform-dependent factors on quantitative image analysis. In a study of quantitative image analysis errors arising from platform-dependent image scaling, Chenevert et al. (4) used a “variable signal” phantom and an ice-water phantom, using 4 MRI scanners, to acquire pseudodynamic images and apparent diffusion coefficient maps, respectively. The resulting data were analyzed by 8 QIN centers using 16 different SW tools. They found that images generated by one of the scanners incorporated pixel intensity scaling that was not accounted for by 13 of the SW tools tested, and only 3 SW tools were modified to perform image scaling and exhibited proper apparent signal change when comparing data from multiple series of acquisitions. Inconsistencies in image scaling measures among imaging platforms may lead to errors when comparing imaging data in multicenter clinical trials. In conclusion, the authors recommended corrective actions for image scaling to be taken by manufacturers and the imaging research community.

Precision and accuracy in tumor segmentation are important aspects of quantitative imaging that may have a significant impact on downstream data analysis, treatment planning, therapeutic dose delivery, and response evaluation. QIN investigators have engaged in multicenter evaluations of image segmentation algorithms applied to clinical images and phantoms. In a recent QIN multi-institutional study, Kalpathy-Cramer et al. (5) conducted a challenge to assess an algorithm bias in the repeatability and reproducibility of nodule segmentation and volume estimation in CT images of lung cancer from 40 patients and a phantom containing 12 nodules of known volumes, using algorithms developed at 3 participating institutions. They found a higher statistically significance agreement in spatial overlap between segmentations generated by multiple runs of the same algorithm than segmentations generated by different algorithms (*P* < .05) and higher spatial overlap of segmentations on the phantom nodules (*P* < .05). They also found that algorithms with the highest accuracy in nodule volume estimation were not the most precise (repeatable), and considerable variations in algorithm performance was observed, particularly on a subset of heterogeneous nodules. They asserted that their results underscored the need for assessing algorithm performance on clinical data in addition to phantom data, and they recommended that the same SW tool be used at all time points in longitudinal studies. Given that the study used a small number of nodules, the authors could not draw conclusions about the relative performance of the algorithms used, but they suggested that their study provided precise methods for segmentation algorithm comparison and sources of variability and their manifestations.

Multicenter analytical studies such as those described above may be performed most efficiently in a network setting, where member teams have access to advanced resources and expertise and share common overarching goals with respect to the network mission, technical developments, and clinical needs. In light of the potential value of challenges for QIN, in 2015, the QIN Executive Committee, composed of the network's principal investigators, recommended the formation of a Challenge Task Force (CTF) to develop policy guidelines to better streamline the challenge process and garner the potential value of network-wide activities. The QIN CTF included the Chair of the QIN Executive Committee (Nola Hylton, PhD), representatives from each QIN Working Groups (John Buatti, MD; Tom Chenevert, PhD; Jayashree Kalpathy-Cramer, PhD; Daniel L Rubin, MD, MS; and John J. Sunderland, PhD), and selected NCI program staff (Keyvan Farahani, PhD and Robert J. Nordstrom, PhD). Recommendations made by the CTF were reported to the QIN Executive Committee and were endorsed by the same. This article presents the recommendations of CTF for processes and best practices for performing QIN-wide challenges, project prioritization and oversight, reporting and dissemination of the results.

## Methods

The CTF conducted a survey of all QIN challenges to date (as of October 2015). Table 1 provides a list of the challenges and brief descriptions. After careful consideration of the type of activity performed in each project, CTF identified 2 distinct types of projects conducted by the network. Because of the specific task performed in each project and methods for participation and evaluation of the results, CTF considered it important to categorize these activities into computational challenges (or challenges for short) and collaborative projects, as defined below.

### 

#### QIN Computational Challenge.

A multisite test of computational algorithms designed to perform quantitative image processing and/or analysis for a given task, with direct technical or clinical relevance to QIN projects, using designated training and test data sets, relevant physical or clinical reference standards, and evaluation metrics. QIN challenges may be further divided into the following subcategories:

(1) *Technical Challenge*: Testing performance characteristics of algorithms based on physical standards and metrics (eg, image markup, spatial or functional accuracy, and repeatability). A technical challenge may test the performance of a tool or a method deployed in a specific technical task (eg, tumor segmentation). The immediate outcome of a technical challenge would be a set of tools, or a class of methods, for technical assessment, and the resulting annotations or other processed data. The lung CT segmentation (5) and the fluorodeoxygloucose PET segmentation challenge (results submitted for publications) (Table 1) are examples of technical challenges.
(2) *Clinical Challenge*: Testing performance characteristics of algorithms based on clinical standards or criteria for clinical decision support in evaluation of response to therapy. A clinical challenge may test performance of a tool or a method, such as evaluation of an imaging biomarker, having a direct connection with the clinical decision-making process. The immediate outcome of a clinical challenge would be a set of benchmarked algorithms, SW tools, or imaging biomarkers for quantitative imaging in predicting or evaluating response to therapy.

#### QIN Collaborative Project.

An analytical study of tools, techniques, scientific and clinical parameters, and protocols, or otherwise, an opinion survey of members sharing similar goals.

Cataloging the outcomes of such projects may provide a useful resource to current and future members of the QIN, NCI, and the greater scientific research community. Based on this definition, several past challenges performed in QIN fall in the category of collaborative projects (2–4).

Key resources for conduct of Challenges and Collaborative Projects (CCPs) include shared data archives and platforms for computational evaluation (for challenges) and collaborative analysis (for collaborative projects). There are several resources currently available to the QIN community to facilitate the conduct of CCPs. These include the Cancer Imaging Archive (www.cancerimagingarchive.net) for sharing of large image data sets; QINLabs, an SW environment, developed by Kalpathy-Cramer et al., based on CodaLab, an open source challenge evaluation platform (Microsoft Research Inc., Redmond, Washington); and the National Cancer Informatics Program (NCIP) HUB (https://nciphub.org). Based on the Hubzero™ platform (6), NCIP HUB, managed by the NCI Center for Biomedical Informatics and Information Technology (CBIIT), is a resource for collaboration and sharing of data and SW tools by the cancer research community. QIN members have access to these resources and are encouraged to use them in conducting their network-wide projects.

**Figure 1.**
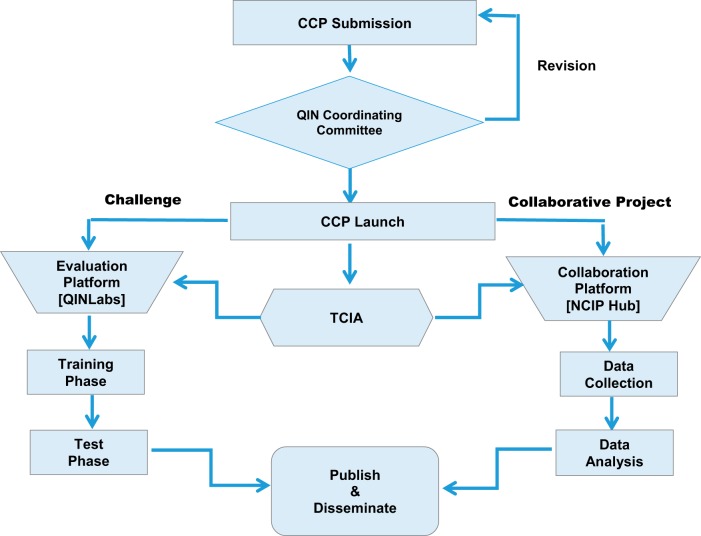
A flowchart of Quantitative Imaging Network (QIN) processes for conducting Challenges and Collaborative Projects (CCPs). The Cancer Imaging Archive (TCIA), QINLabs, and National Cancer Informatics Program (NCIP) HUB are resources available to QIN members to share data, run challenges, or conduct collaborative projects, respectively.

**Figure 2.**
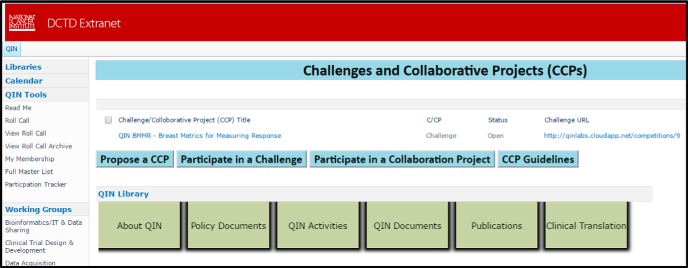
The CCP panel on the QIN SharePoint site serves as a bulletin board for information about current QIN CCPs.

The QIN CTF set forth the policy and processes for performance of CCPs by network members. Figure 1 shows a flow diagram for CCP processes as currently implemented. In QIN, the CCP organizer may be an individual team member working with, or independent of, a QIN Work Group, although in most cases CCPs are developed through Work Groups. When proposing a CCP, the use of standard scientific definitions, terminologies, data types, and metrics is encouraged. The CCP application form may be accessed and submitted through a dedicated module in the NCI QIN SharePoint site (Figure 2), accessible only by QIN members. A CCP proposal outlines the specific aim of the project, its relevance to the QIN mission, and data or methods to be used, including evaluation metrics (for a challenge) and analytic methods (for a collaborative project). The QIN Coordinating Committee, composed of the chairs of QIN Work Groups and NCI program staff, will review CCP proposals during the committee's monthly conference calls. The committee will review and evaluate each application for completeness, alignment with the goals of the most recent QIN Program Announcement, and technical or clinical priorities in support of quantitative imaging in oncology. The Coordinating Committee may accept a CCP proposal or recommend revisions to improve the quality of the proposal. Active CCPs are announced to the network through the QIN SharePoint site and through group e-mails.

**Figure 3.**
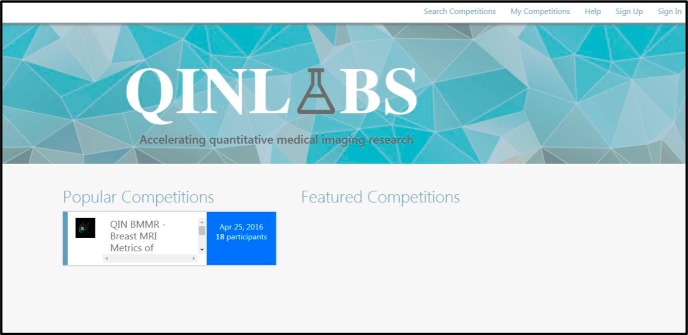
QINLabs provides a customizable platform for evaluation of computational challenges with participation of QIN member sites.

Applicants are encouraged to use The Cancer Imaging Archive (TCIA) as the image repository for CCPs, and in case of challenges, properly designate the “training” and sequestered “test” data sets. Although, at times, an existing image collection on TCIA may provide a valuable data set for a challenge competition, applicants are cautioned that preexisting public access to the data set may compromise its value for use as a test set in a challenge competition. In such rare cases, applicants are required to describe how they handle use of publicly available data for a challenge while preserving the integrity of the competition. One option for the organizers of a challenge may be to supplement the public data set with a private data set that has comparable image attributes and quality. Once a CCP is approved, the organizer may work with the relevant evaluation or collaboration platform team to prepare for hosting their project on that platform (QINLabs (Figure 3) and NCIP HUB for Challenges and Collaborative Projects, respectively). This will include development of a customized user interface page required for each CCP. In the case of a challenge, the organizer will also work with TCIA (or another qualified repository) to deposit the designated training and test data sets before the start date.

As shown in Figure 1, the execution of each CCP includes 2 phases. In case of a challenge, these are the training and the test phases, and in case of a collaborative project, these are the data collection and the data analysis phases. In general, it is recommended that these phases be conducted over a 2-month period, but the network exercises some flexibility in the CCP timeline to allow for better preparation for each phase or more participation. Participation in CCPs is open to all QIN members, both full and associate members. QIN associate members are independent federally funded national or international researchers with project aims relevant to the QIN mission, and approved by the QIN Executive Committee.

**Figure 4.**
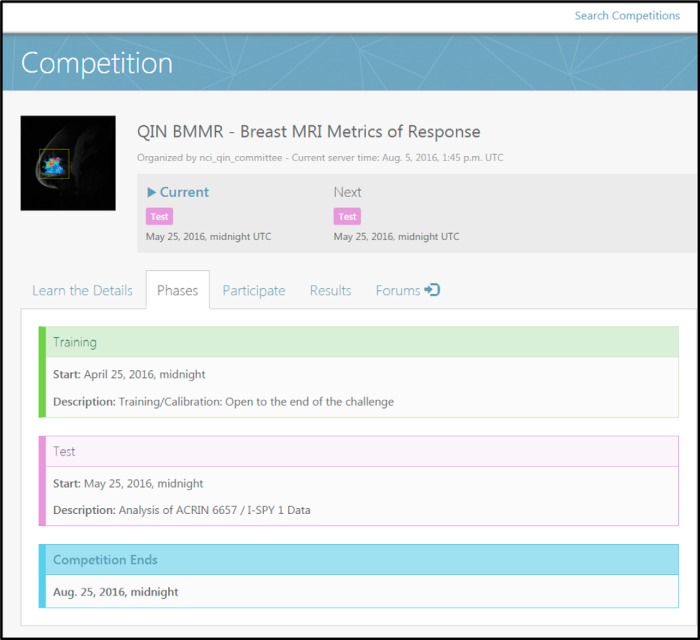
Screenshot of a QINLabs page for the Breast MRI Metrics of Response (BMMR) clinical challenge. Participants can obtain general information about a challenge and its various phases, participate in the challenge, view current results, and post questions to the forum.

The results of CCPs will be based on the proposed “evaluation metrics” or criteria suggested by the CCP originator at the outset, and the ranking of the results will follow such criteria. The host platform (QINLabs or NCIP Hub) will keep a record of various phases (Figure 4), the results and products of each CCP. In case of challenges, it is expected that segmentations, annotations, or other artifacts of the challenge data will be deposited back in the original repository (eg, TCIA) and assigned a Digital Object Identifier (DOI). In case of collaborative projects, the analytical results will be deposited in the NCIP Hub and DOIs will be assigned.

Upon completion of a CCP, the organizers are expected to prepare a concise report summarizing the activity, outlining any issues, and the results. The report will describe plans for future dissemination of the CCPs through scientific societies and/or the results through peer-reviewed scientific publications. These reports will be presented at the annual meeting of QIN and captured in the QIN Annual Reports. The CCP organizer will lead the effort for preparation of a manuscript for submission to a peer-reviewed journal, describing the CCP and its results (in case of challenges), or a consensus report (in case of collaborative projects). Such journal publication will include all key participants as coauthors and DOIs reference to archived data sets and products of the CCP.

The QIN Coordinating Committee, in collaboration with the QIN Executive Committee and NCI program staff, will provide an oversight to QIN CCPs. This will include review and prioritization of proposals, recommendations for amendment of proposed projects before their execution, monitoring of the process, and review of final reports and relevant communications with the QIN Executive Committee. In case of any dispute or other issues about the execution or outcome of a CCP, the matter will be referred to the Coordinating Committee that will work with the program staff and, if necessary, the QIN Executive Committee to resolve such matters. Disputes between applicants and the Coordinating Committee will be referred to the QIN Executive Committee for resolution.

## Discussion

Advances in computational power together with online access to large data sets have ushered in an era of community-based challenges and crowd-sourced projects that address a wide range of scientific and social issues (7–12). The field of medical imaging is especially well positioned to take advantage of challenges and community-sourced solutions. Over the past several years, there has been an increase in the application of challenges to benchmark algorithms for specific clinical or technical tasks, including detection, registration, and segmentation (13–16).

CCPs provide efficient means for the QIN to engage network members in assessment of SW tools and analytical solutions that address various aspects of image acquisition and analysis or clinical decision support processes. CCPs are particularly useful in a network setting, as they provide a vehicle for QIN to address many overarching scientific problems in a collaborative fashion and help harness the power of the network. They promote, and require, preparation of well-curated data sets for multicenter analysis, benchmarking of quantitative tools, comparison of methods, development of consensus on approaches to quantitative imaging in oncology, and promotion of best practices. However, having a network engaged in collaborative work does not guarantee desirable outcomes, particularly when teams are involved with multiple ongoing network-wide projects. The overall mission of the QIN CTF is to develop policies and procedures designed to harmonize and streamline the prioritization, execution, and dissemination of results from CCPs, and leverage available resources toward their accomplishment. Some of the resources used in design and execution of CCPs are supported through other NCI initiatives. These resources include TCIA (www.cancerimagingarchive.net), the NCI Informatics Technology for Cancer Research (ITCR) (http://itcr.nci.nih.gov/); the Center for Bioinformatics and Information Technology (CBIIT) (https://cbiit.nci.nih.gov/); and imaging data from clinical trials completed through ECOG-ACRIN (http://ecog-acrin.org/), an NCI cooperative group that was formed by the merger of the Eastern Cooperative Oncology Group (ECOG) and the American College of Radiology Imaging Network (ACRIN).

Over the next several years, imaging data from 14 ECOG-ACRIN clinical trials will be made available on TCIA, initially to QIN investigators and after a period of 1 year to the general public. QIN investigators will have opportunities to use these data sets from ECOG-ACRIN trials in constituting a range of CCPs. The first set of imaging clinical trial data provided by ECOG-ACRIN that is undergoing this process is the MRI data from ACRIN trial 6657. The ACRIN 6657 trial tested contrast-enhanced MRI for the ability to predict pathological response and recurrence-free survival for patients with stage II or III breast cancer receiving neoadjuvant chemotherapy (17). A recently conducted QIN Challenge, titled “Breast MRI Metrics of Response (BMMR),” based on the ACRIN 6657 data, had the following 2 aims: identify imaging metrics (predictors) derivable from contrast-enhanced breast magnetic resonance images acquired in the ACRIN 6657 trial that show statistically significant association with recurrence-free survival and demonstrate improvement in predictor performance over functional tumor volume, the primary imaging variable tested in ACRIN 6657. The BMMR Challenge is an example of a clinical challenge, one which will help benchmark algorithms developed by participating QIN teams in the identification of new imaging biomarkers that may provide improvements in predicting response to neoadjuvant chemotherapy in breast cancer. It is expected that the ACRIN trial 6657 imaging data will soon become public, at which point, the BMMR organizers plan to conduct a collaborative project on the entire data set.

The strategy of conducting both a challenge and a collaborative project on a data set, exemplified by the BMMR, may prove beneficial in the development of benchmarks and in reaching a consensus on methods using the same public data set. Moreover, publication of reports from such paired CCPs, along with public access to the related clinical imaging data through TCIA, should provide invaluable resources to the research community, where researchers may be able to compare the performance of their SW tools with the performance of those developed and tested by QIN. Future steps in the QIN CCP initiative, currently at an early exploration stage, include cataloguing of SW tools and consensus documents, developed through QIN CCPs, and open access to such tools and documents. Basic and clinical science researchers would potentially be able to adapt QIN tools and consensus opinions in prospective clinical trials or further advance the tools.

Development of SW tools for quantitative imaging in cancer, consistency in accuracy and precision of imaging methods across commercial clinical systems, and the relevant clinical advancement of quantitative imaging, are among the major goals of the NCI QIN. CCPs provide the means for QIN to advance toward these goals through cooperation of network members. The CCP policies and processes developed by the QIN CTF provide the necessary goalposts to help QIN members conduct CCPs in a streamlined and transparent manner and publicly disseminate the results.
